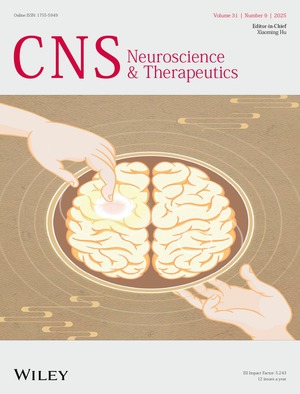# Front Cover

**DOI:** 10.1111/cns.70624

**Published:** 2025-09-28

## Abstract

The cover image is based on the article *Visuomotor Training to Enhance Proprioception of Contralateral Wrist Based on the Cross‐Transfer Effect* by Yizhao Wang et al., https://doi.org/10.1111/cns.70504.